# Topical Insulin as an Add-On Therapy for Leg Ulcer: A Case Report

**DOI:** 10.7759/cureus.39822

**Published:** 2023-06-01

**Authors:** Yusuf Can Edek, Elif Çalışkan Güneş, Hale Nur Ertugay Aral, Esra Adışen, Ahmet Burhan Aksakal

**Affiliations:** 1 Dermatology, Gazi University, Ankara, TUR

**Keywords:** chronic venous leg ulcers, topical agent, ulcer, treatment, insulin

## Abstract

Leg ulcers affect millions of people worldwide, and are a significant cause of morbidity and mortality. Various factors can be etiological agents of leg ulcers such as vascular, neuropathic, infectious, and traumatic factors. Treatment of the leg ulcer can be difficult in some cases despite using different systemic treatments and local wound care, but various newly defined treatment modalities are discussed in the literature and topical insulin application is one of them. Insulin is a hormone that is essential for regulating blood glucose and lipid levels, also insulin can have local effects when applied topically. Various mechanisms like regulation of inflammation, collagen synthesis, and angiogenesis have been explained to understand topical insulin's effects on the wound. There are case reports and studies on the usage of topical insulin on diabetic ulcers and decubitus ulcers. We applied topical insulin as an add-on therapy on a treatment-resistant leg ulcer and observed the healing of the lesion. The use of topical insulin as an add-on therapy may reduce treatment time and speed up wound healing. Topical insulin can be considered as an additional therapy for treatment-resistant ulcers.

## Introduction

Insulin is an essential hormone for regulating blood glucose and lipid levels [1]. Besides its systemic effects, many reports indicate that insulin has local effects when applied to the skin. Many studies and case reports have been published on the use of topical insulin treatment for various diseases, including dermatological, surgical, ophthalmological, and others [2-4]. Systemic insulin treatment may have a healing effect on wounds, but its side effects, such as hypoglycemia and hypokalemia, may limit its use. When topical insulin is applied to the skin, it yields the same beneficial effects with the advantage of the absence of systemic side effects and also is relatively low cost [2]. Although the mechanism of topical insulin's effect on the wound is unknown, various pathways have been described to understand the mechanism. Herein, we report our experience in treating venous stasis ulcers with topical insulin as an add-on therapy.

## Case presentation

**Figure 1:**
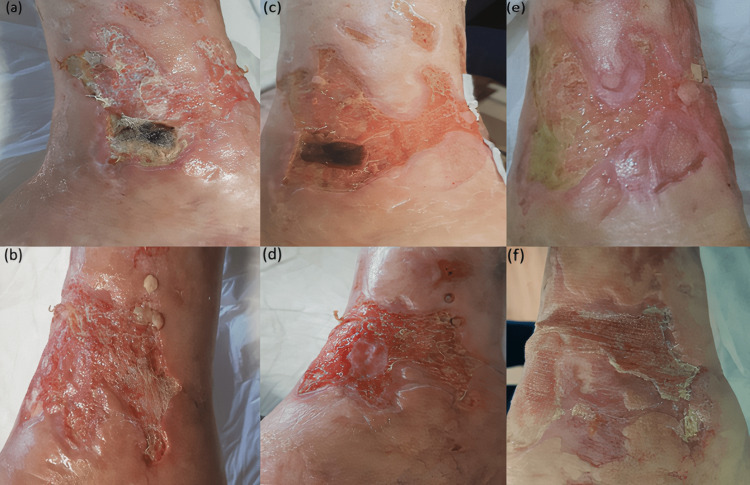
(a), (b) ulcer on the left leg before topical insulin treatment, (c), (d) one week after the treatment, (e), (f) four weeks after the treatment.

A 64-year-old man presented to our clinic with a leg ulcer. His medical history revealed he had type 2 diabetes on metformin, hypertension on valsartan hydrochlorothiazide, chronic venous insufficiency, and was also an active smoker. The lesion started seven months ago on the left leg, according to the patient’s statement. He used topical-systemic antibiotic treatments, which were ineffective. When the lesion became larger, he came to our clinic. A dermatological examination revealed an ulcer on the left leg above the ankle, measuring 9x6 cm in diameter, with irregular borders and fibrin tissue on the lesion (Figure 1). Histopathological analysis of punch biopsy taken from the edge of the ulcer was compatible with changes secondary to venous stasis. The ulcer was accepted as a venous stasis ulcer after clinical, histopathological, and Doppler-USG examinations. When wound care therapies such as compression therapy, leg elevation, ulcer debridement, and wound dressing proved ineffective, after consulting case reports of using topical insulin on diabetic ulcers and decubitus ulcers, we added topical insulin to leg elevation and ulcer debridement treatments. Daily 1 ml insulin (Human Actrapid insulin 40IU/ml®) was sprayed over the ulcer by an insulin injector once daily in the hospital and was covered with sterile gauze. Blood glucose levels were measured before and after the application and no abnormalities were observed. The ulcer started to heal after the first week, and by the fourth week, significant improvement (75% decrease in ulcer area) was observed in the ulcer (Figure 1).

## Discussion

Leg ulcers are prevalent worldwide, and are a significant cause of morbidity and mortality. The etiology of leg ulcers includes vascular, neuropathic, infectious, and traumatic factors. Venous ulcers are the most commonly seen type of leg ulcer [5]. While the treatment of the leg ulcer can be challenging in some cases despite using different systemic treatments and local wound care, various newer treatment modalities have been discussed in the literature, including topical insulin application [2].

Acceleration of healing of our patients’ treatment-resistant ulcers supports the beneficial effect of topical insulin on wound healing. Pawar et al. used topical insulin on treatment-resistant erosions and ulcers in a pemphigus vulgaris case in addition to systemic treatment and observed healing of the lesions [6]. Additionally, a case with acne scars was reported in which topical insulin was applied by micro-needling, and the patient's condition improved [7]. In addition to dermatological diseases, there have been case reports of applying topical insulin on corneal ulcers [4].

Insulin shows its effects by regulating the inflammatory response and wound-healing process. Increased M2-macrophages, interleukin-10 (IL-10) levels, and altered macrophage functions, including chemotaxis and phagocytosis are some of the mechanisms underlying insulin's anti-inflammatory and wound-healing effects. Insulin can induce the migration of vascular endothelial cells, keratinocytes, and fibroblasts via the PI3K-Akt-Rac1 signaling pathway. In addition to cell migration, insulin increases the production of VEGF and TGF-ß1 synthesis, which have significant roles in angiogenesis and the formation of granulation tissue. Insulin also affects the synthesis of collagen tissue by modulating collagen maturation and organization [2,8-10].

In a randomized controlled study by Chen et al., they applied topical insulin to one group and saline to the other group for ulcer treatment. It has been observed that topical insulin treatment is effective in promoting wound healing by regulating macrophage infiltration, increasing MCP-1 expression, phagocytosis, and inflammatory cytokine release [8]. As a result of these effects, insulin plays an important role in all parts of wound healing.

The use of topical insulin as an add-on therapy may reduce treatment time and speed up wound healing. We had a remarkable clinical outcome in our case by combining topical insulin therapy with systemic treatments. We believe topical insulin application should be considered as an alternative treatment for non-healing ulcers. Currently, there is not enough data to define which leg ulcers respond to topical insulin therapy. Thus, future research and case reports may be useful in determining the efficacy of topical insulin treatment for various kinds of dermatological conditions.

## Conclusions

Leg ulcers are a significant cause of morbidity and mortality and various factors can be etiological agents of leg ulcers including vascular, neuropathic, infectious, and traumatic factors. Treatment of the leg ulcer can be difficult in some cases despite using different systemic treatments and local wound care. Topical insulin application is a newly defined treatment method that can be used when dealing with treatment-resistant ulcers. Insulin works by regulating the inflammatory response and wound-healing process. Here, we report a case of a 64-year-old man who got a leg ulcer due to venous stasis and was treated with topical insulin treatment. With this case, we want to emphasize that topical insulin application may be one of the promising treatments for leg ulcers.
